# Endohedral metal-nitride cluster ordering in metallofullerene–Ni^II^(OEP) complexes and crystals: a theoretical study†

**DOI:** 10.1039/c9cp00634f

**Published:** 2019-04-17

**Authors:** Vasilii Dubrovin, Li-Hua Gan, Bernd Büchner, Alexey A. Popov, Stanislav M. Avdoshenko

**Affiliations:** a IFW Dresden, Helmholtzstraße 20, 01069 Dresden, Germany; b School of Chemistry and Chemical Engineering, Southwest University, Chongqing 400715, China

## Abstract

The ordering of endohedral clusterfullerenes Sc_3_N@C_80_ and YSc_2_N@C_80_ co-crystallized with Ni(OEP) and isolated complexes with Ni(OEP) have been investigated theoretically. Having used multiple orientations of M_3_N clusters inside the cages with Fibonacci sampling, we describe the effect of intermolecular interactions on the orientation of the endohedral cluster.

Molecules of fullerenes encapsulating metal atoms or clusters (*aka* endohedral metallofullerenes, EMFs^1^) can be considered as molecular ball-bearings or gyroscopes consisting of two semi-independent structural units. Despite the strong interactions between the metal atoms and fullerene π-system, the potential energy surface for the rotation of the clusters inside the cages is often rather shallow, leading to quasi-free rotations of endohedral species. A prominent example is found in nitride clusterfullerenes M_3_N@C_80_-*I*_h_, where M can be Sc, Y, lanthanides, or any combination of them.^2^ The icosahedral C_80_ cage is almost a sphere with many equivalent bonding sites for metal atoms. NMR spectroscopy confirms that in solutions the M_3_N cluster rotates freely at room temperature.^3^ For Sc_3_N@C_80_, early computational studies have predicted the barrier to this rotation to be below 10 kJ mol^−1^,^4^ and similar estimations have been made based on STM^5^ and NMR measurements.^6^ Obtaining ordered assemblies of such molecules in crystals or in layered structures on surfaces is a challenging task, which, however, may have a high practical impact because the orientation of endohedral clusters affects the electronic and magnetic properties of EMF materials. Furthermore, molecular structure determination by single-crystal X-ray diffraction is complicated by a severe disorder caused by a loosely bound endohedral cluster and rotation of the fullerene cage itself.

Early in the days it was noticed that empty fullerenes would not rotate as freely if they were captured in non-covalent complexes as co-crystals.^8^ This way a precise molecular structure of many fullerenes got to be known with a little to no disorder.^9^ In modern EMF research, co-crystallization of EMFs with metal-octaethylporphyrin (Me^II^(OEP), Me^II^ = Ni or Co)^10^ has become a *de facto* standard for X-ray diffraction studies.^11^ For nitride clusterfullerenes M_3_N@C_80_-*I*_h_, it turns out that not only has the cage been subdued to order by the presence of Me(OEP), but also the inner nitride clusters.^12^ It was also found that the orientation of the M_3_N cluster is very sensitive to its exact composition – be it monoionic Sc_3_N or a mixed-metal LnSc_2_N system with Ln = La, Ce, Gd, Tb, Er, or Dy.^7,13^ Taking into account a very low barrier of inner rotation for the M_3_N clusters, this is a remarkable observation of great importance in EMF chemistry. Although the ordering effect has already been known for years, it would be fair to say that its origin is still not well understood. However, it would be of great use to know the exact energy scale of the effect and if it has a local or cooperative nature to strategize the search for and design of EMF materials as the ability to form ordered arrays is an important criterion.

In this communication, we explore the influence of the interaction between M_3_N@C_80_ and Ni(OEP) on the orientation of the endohedral cluster by comprehensive computational studies. To study the EMF–Ni(OEP) interaction in different environments, we design two sets of systems: (I) co-**CR**ystals (**CRS-*x***, *x* = 0, 1) of Y_*x*_Sc_3–*x*_N@C_80_ with Ni(OEP) in the experimental crystal settings taken from ref. 7 (Fig. 1a) and (II) **CO**mplex **S**et (**COS-*x***, *x* = 0, 1) with the isolated Y_*x*_Sc_3–*x*_N@C_80_·Ni(OEP) molecular complexes. Since the ionic radius of Y is close to those of a set lanthanides (Tb, Dy, Ho, *etc.*), the results obtained in this work for YSc_2_N@C_80_ are representative of a larger group of MSc2N@C_80_ compounds, including those with magnetic lanthanide atoms. Computations were done using the VASP5.0 code at the PBE-D3/PAW level of theory with a plane-wave basis cut-off of 400 eV consistent with the pseudopotential.‡^14^

The endohedral cluster in the isolated M_3_N@C_80_-*I*_h_ molecule has 2–3 energy minima with small energy differences. Obviously, the interaction of the EMF molecule with Ni(OEP) changes this situation, else the ordering effect would not be observed experimentally. In other words, the potential energy surface (PES) for the cluster inside the cage becomes more prolific when it interacts with Ni(OEP) with the appearance of pronounced orientational minima. But since EMF–Ni(OEP) interactions are non-bonded, the energetic effect on the endohedral cluster cannot be very strong. To make the theoretical depiction of the systems complete and consistent, it is necessary to ensure that all energy minima corresponding to the different orientations of the cluster inside the fullerene are found. It would be insufficient to optimize one particular structure with an arbitrary orientation of the endohedral cluster. As illustrated schematically in Fig. 1b, for the complex PES as a function of the order parameter *Θ*, with multiple energy minima, a single optimization will most probably proceed into the closest energy minimum in the configurational space (Fig. 1b, the green dot and trajectory line), and not necessarily into the global energy minimum. Only if the configurational space is properly sampled (Fig. 1b, all starting points and trajectories), the reliable reconstruction of the PES minima is feasible. Exhaustive and regular sampling of the orientational phase space is achieved here by the Fibonacci sphere sampling. Namely, for each system 120 conformational isomers were generated by rotating the internal cluster along the Fibonacci sphere with respect to the nitrogen as shown for **CRS-0** in Fig. 1. Herewith one of the Sc atoms in Sc_3_N@C_80_ or the Y atom in YSc_2_N@C_80_ walks over the sphere, while the M_3_N cluster is kept rigid through relevant rotation in polar coordinates (see the ESI† for generation script). The angle between the M–N bond and the *Z*-axis defines the order parameter *Θ* (Fig. 1). Since each conformer tends to relax to the closest minimum, insufficient and/or biased sampling may lead to unfair judgments about the energy minima distribution. The proposed homogenous sampling ensures maximum conformation space coverage for a given number of nodes.

At first, the outlined computational approach is applied for the isolated Sc_3_N@C_80_ molecule. Fig. 2a shows the energy distributions of the optimized conformers. Optimization of all 120 initial structures generated by Fibonacci sampling results in only two real minima with *C*_3_ and *C*_s_ symmetry (see Fig. S1 of the ESI†) in agreement with earlier DFT studies.^4^ The energy difference between the two structures is only 2.3 kJ mol^−1^, which is consistent with the free rotation of Sc_3_N within C_80_ at room temperature. For an isolated YSc_2_N@C_80_ molecule, the procedure resulted in 3 unique structures (Fig. 2d, and Fig. S2 of the ESI†) with relative energies of 0, 2.6, and 3.8 kJ mol^−1^.

For the **CRS** and **COS** groups (Fig. 2), the energy distributions of the optimized structures still have a narrow energy spread (Δ*E* ≤ 16 kJ mol^−1^). However, these narrow ranges are visibly larger than those in the isolated Sc_3_N@C_80_ and YSc_2_N@C_80_ molecules, which illustrates the effect of intermolecular interactions on the orientation of the M_3_N cluster. The most stable structures in all cases are consistent with experimental observations. For example, in the **CRS-1** and **COS-1** systems, the Y atom is placed remotely to the Ni-site. However, the energy differences of energetically close conformers are minuscule. Therefore, in the window of 1*k*_B_*T* at room temperature (2.5 kJ mol^−1^), one would find up to 10 conformers (as in the case of **CRS-0**). Nevertheless, structurally, these minima are very similar to the global minimum.

Let’s consider the **COS** and **CRS** groups in more detail. When the M_3_N@C_80_ molecule is set to interact with Ni(OEP), the energy distribution of the optimized structures changes considerably. Instead of 2–3 unique minima found for the isolated M_3_N@C_80_ molecules, calculations predict the appearance of multiple different minima in the **COS-0,1** structures. This shows that the non-covalent interaction with Ni(OEP) breaks the orientation isotropy of the icosahedral fullerene cage. Second, the Δ*E* spread of the **COS** structures (~12 kJ mol^−1^) is increased four-fold in comparison to the isolated Sc_3_N@C_80_ and YSc_2_N@C_80_ molecules. These differences between M_3_N@C_80_ molecules and **COS** systems clearly point to the considerable effect of the intermolecular interaction on the orientation of the endohedral cluster in M_3_N@C_80_·Ni(OEP) complexes.

The lowest energy conformers in **COS-0,1** are grouped near *Θ* = 0, which corresponds to the vertical orientation of the Sc–N or Y–N bond remotely from the Ni(OEP)-coordination site of the fullerene cage (Fig. 2c and d). Two other Sc–N bonds are oriented towards the Ni(OEP) molecule. This orientation of the endohedral cluster corresponds to the experimentally observed orientation in M_3_N@C_80_·Ni(OEP) crystals.^7,13^

The cluster composition plays an important role in the overall energy distribution. Since the Sc–N–Sc angle is close to 2π/3, stable **COS-0** conformers can also be obtained for *Θ* = 2π/3 (in this case another Sc atom has a vertical orientation). Indeed, clustering of low-energy conformers can be seen near *Θ* = 2π/3. In total, the energy distribution of the **COS-0** conformers is very dense, giving only a moderate ordering effect for the energy cut-off of 1*k*_B_*T* at room temperature. For the YSc_2_N cluster in the **COS-1** system, the situation is different. The conformer with *Θ* = 0 corresponds to the well-defined global minimum. If the positions of Y and one Sc atom are flipped (giving *Θ* = 2π/3), the energies increase by *ca.* 5 kJ mol^−1^. Thus, the ordering effect for YSc_2_N@C_80_·Ni(OEP) is more pronounced and gives essentially one structure for the energy cut-off of Δ*E* = 2.5 kJ mol^−1^.

When M_3_N@C_80_·Ni(OEP) complexes are placed in a crystalline environment, the energy distributions in the **CRS-0,1** systems resemble those of **COS-0,1**, but with noticeable alterations. First, the relative energy spread is increased further by *ca.* 25–30%. Second, the stability of the conformers near *Θ* = 0 and *Θ* = 2π/3 is enhanced in **CRS-0** compared to **COS-0**. Thus, the ordering effect on the Sc_3_N cluster is stronger in the crystal than in the isolated Sc_3_N@C_80_·Ni(OEP) complex. Third, for **CRS-1** the crystalline environment enhances the relative stability of the conformers with *Θ* = 2π/3. The conformer with the vertical orientation of the Y–N bond (*Θ* = 0) is still the most stable, but the structure with the flipped positions of Y and Sc is only 2.5 kJ mol^−1^ higher. Thus, the crystalline environment enhances the preferential orientation of the nitride clusters but makes the Y and Sc positions in YSc_2_N@C_80_ less energetically different. This agrees with our recent study of DYSc_2_N@C_80_·Ni(OEP) crystals,^7^ which showed that the vertical orientation of the Dy–N bond was more preferable, but the site with the flipped Sc and Dy positions had up to ~20% occupancy in one of the crystal batches.

As both Ni(OEP) and EMF molecules have inhomogeneous charge distribution, electrostatic interactions may play an important role. This factor can be analyzed qualitatively with the help of electrostatic potential distribution^15^ as shown in Fig. 3. The nitrogen atoms in Ni(OEP) create a rather high negative potential. To achieve optimal electrostatic interactions, an EMF molecule should be oriented towards Ni(OEP) with its highest positive potential sites. As shown in Fig. 3 for YSc_2_N@C_80_, the poles of the cage have a more negative potential as opposed to the Sc- or Y-coordination sites on the equator, where the potential is more positive. The highest relative energies are indeed found for those structures, in which the pole of the EMF faces Ni(OEP) (*i.e.* the M_3_N cluster is aligned parallel to the Ni(OEP), *Θ* near π/2), whereas in the lowest energy structures two metal atoms of the cluster face Ni(OEP). Note that the positive potential around the Sc-binding sites is slightly higher than that for the Y-binding site (Fig. 3), which may explain why the vertical orientation of the Y–N bond is preferable in YSc_2_N@C_80_·Ni(OEP).

To pinpoint the energetic contributions which govern the effect, we analyze the classical dispersion and Coulomb interactions in the **COS-0** system. The dispersion is evaluated according to Grimme’s model (D3).^14*d*^ Electrostatic interactions were evaluated with absolutely-localized molecular orbital energy decomposition analysis (ALMO EDA) as implemented in the Q-chem4.4 code.^16^ The results are summed up in Fig. 4. The median of the relative dispersion energy in the **COS-0** set is close to 2.5 kJ mol^−1^ with no preference for any particular value of *Θ* (Fig. 4 (G)). Differently, the frozen density Coulomb repulsion energy gives a stronger discrimination of the conformers and is able to indicate a few of the most stable structures. However, not all stable structures would be selected based on this criterion, as it appears from the direct comparison in Fig. 4. This discrepancy comes from more complex interactions and cannot be accounted for within the ALMO EDA model, which focuses on electrostatic effects, *e.g.*, charge–charge, charge–dipole, and charge–induced dipole interactions. Moreover, an additional discrepancy can arise due to the lack of long-range Coulomb interactions in the current ALMO EDA model.

To summarize, DFT calculations at the PBE-D3 level provided a reliable description of M_3_N@C_80_·Ni(OEP) systems and revealed how non-covalent interactions with Ni(OEP) result in a substantial ordering effect for the endohedral cluster in Sc_3_N@C_80_ and YSc_2_N@C_80_ molecules. Assuming thermodynamic control with thermal averaging over 2.5 kJ mol^−1^ (RT), the nitride clusters are predicted to be well ordered. However, the energy scale of this effect is rather narrow, and the ordering will disappear if the thermal energy range covers Δ*E* = 10 kJ mol^−1^. The results on YSc_2_N@C_80_·Ni(OEP) can be generalized to other EMFs with a middle-sized lanthanide in place of Y.

## Supplementary Material

† Electronic supplementary information (ESI) available: Further computational details and optimized coordinates. See DOI: 10.1039/c9cp00634f

SI

## Figures and Tables

**Fig. 1:**
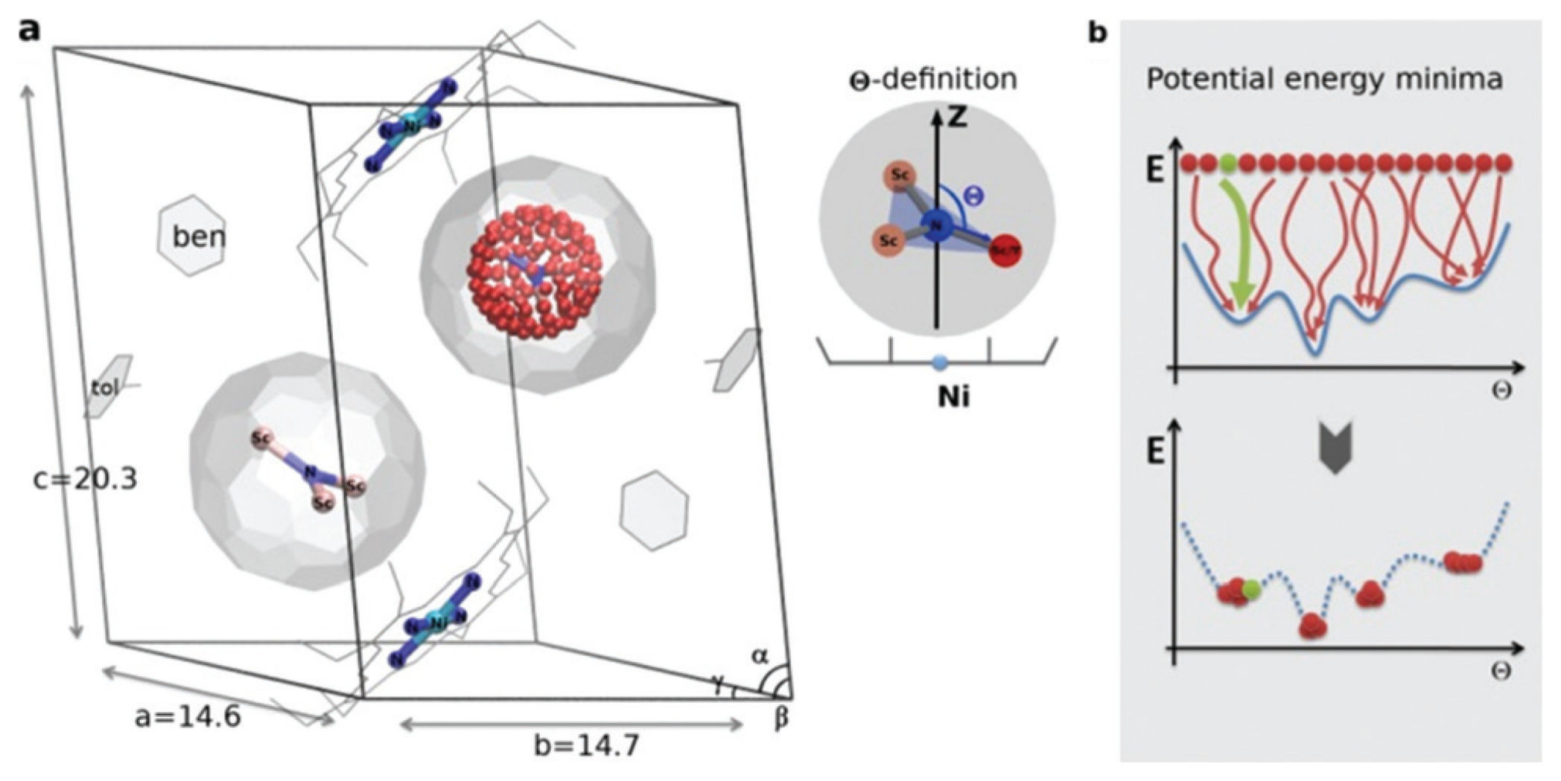
(a) Sc_3_N@C_80_·Ni^II^(OEP) crystal system used in the theoretical study, based on the X-ray data in ref. 7. In one fullerene cage the Sc_3_N cluster is rotated along the Fibonacci sphere with sampling 120-nodes. In the second cage the Sc_3_N cluster has a natural orientation. The order parameter *Θ* is defined as an angle between the atomic position captured in red and the *Z*-axis (black arrow). (b) Schematic illustration of potential energy minima reconstruction based on homogenous sampling (see text for details).

**Fig. 2:**
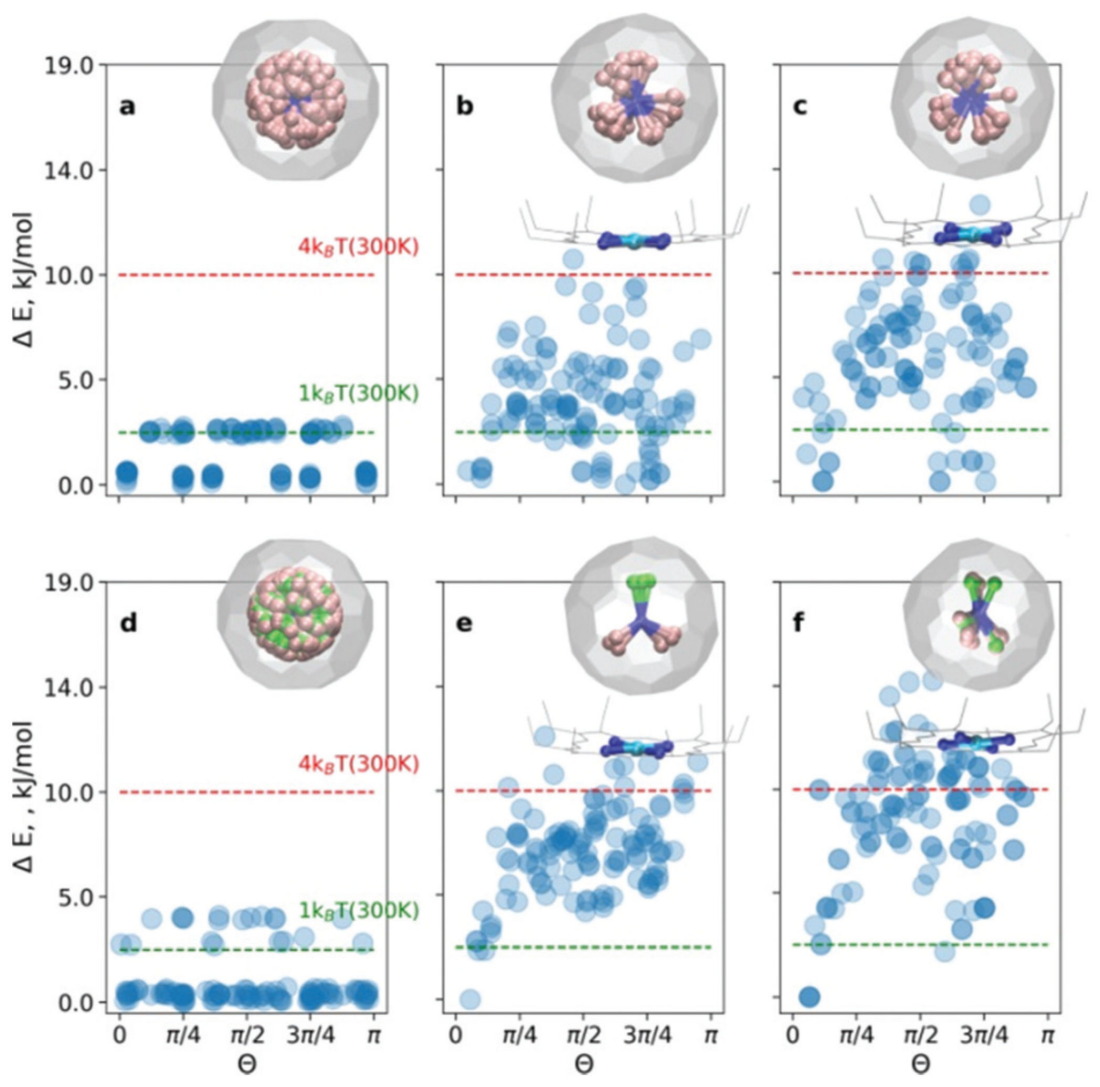
Relative energy distributions as a function of the order parameter *Θ* for (a and d) isolated **Sc_3_N@C_80_** and **YSc_2_N@C_80_**, (b and e) complex systems (**COS-0**, **COS-1**) and (c and f) crystal systems (**CRS-0**, **CRS-1**), respectively. The right upper corner graphics show superposed structures with Δ*E* < 2.5 kJ mol^−1^.

**Fig. 3:**
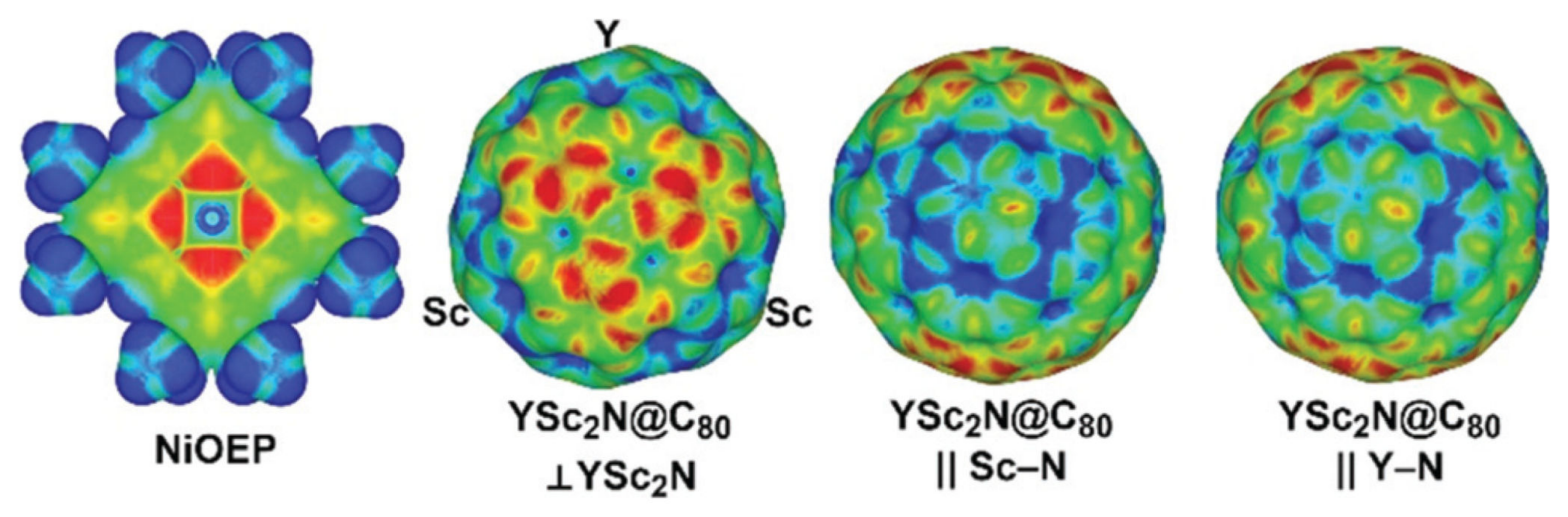
Electrostatic potentials in Ni(OEP) and YSc_2_N@C_80_ molecules (red and blue indicate more negative and positive potentials, respectively) mapped on the electron density isosurface of 0.01 a.u. YSc_2_N@C_80_ is shown in three orientations: normal to the YSc_2_N plane and along the Sc–N and Y–N bonds.

**Fig. 4:**
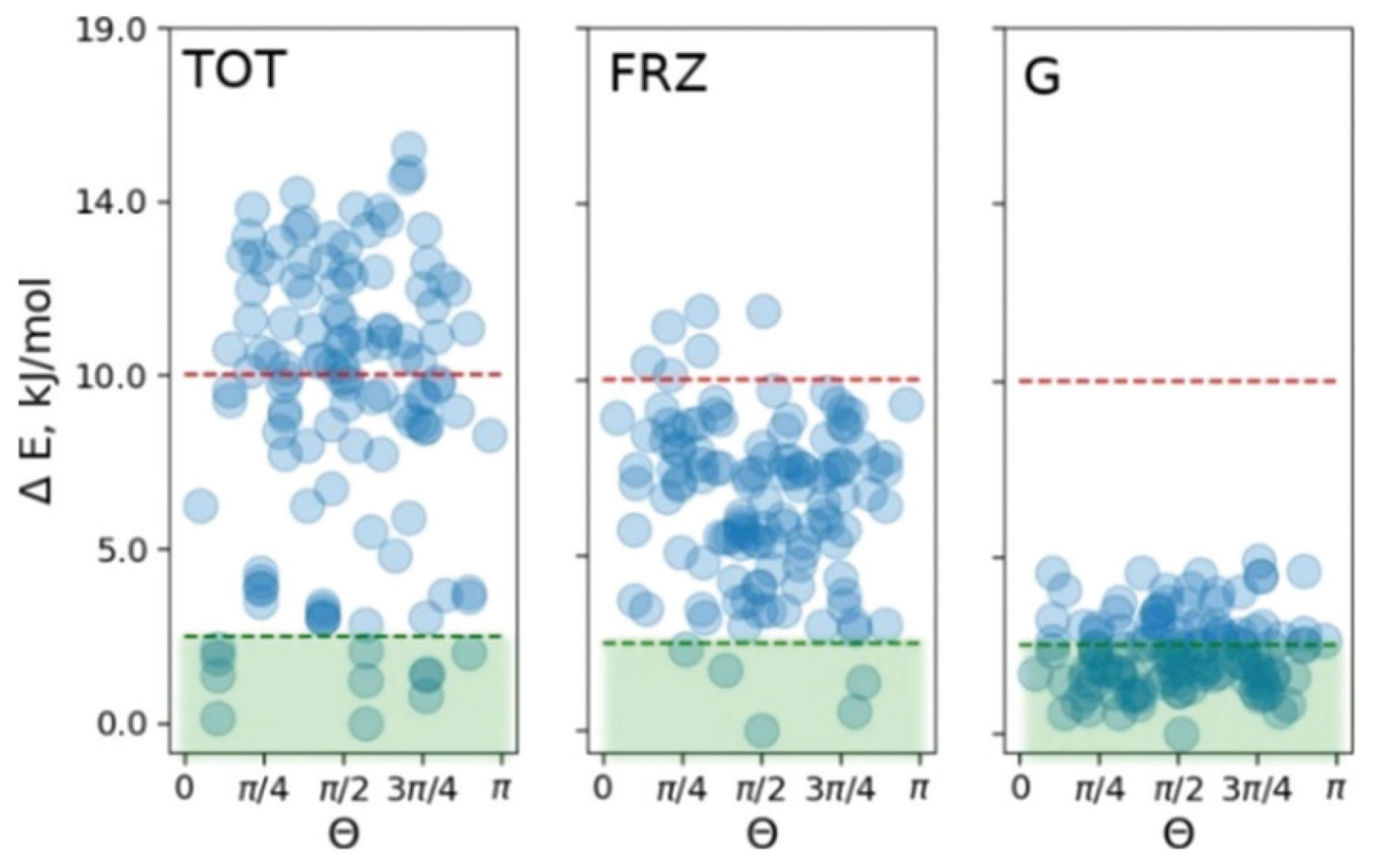
ALMO energy decomposition for the **COS-0** set. TOT, distribution of the total energy at the PBE/6-31G* level of theory; FRZ, frozen density Coulomb interaction energy between Sc_3_N@C_80_ and Ni(OEP) fragments; and G, Grimme’s dispersion energy.

## Footnotes

‡ Each system was optimized to a residual force of 0.005 Å eV^−1^. All optimizations for crystal and molecular systems were performed at the *Γ*-point. The molecular systems were put in a cubic unit cell with a side of 25 Å, which ensures no impact due to interactions with periodic images. The results were analyzed and visualized with in-house Python scripts.^17^ Molecular structures are visualized with the VMD package.^18^
