# Supplementary material for: Endohedral metal-nitride cluster ordering in metallofullerene–NiII(OEP) complexes and crystals: a theoretical study†
Source: Phys Chem Chem Phys. Author manuscript; Available in PMC 2019 Nov 8. (PMC6839963; doi:10.1039/c9cp00634f)
Supplement: SI [file EMS84816-supplement-SI.pdf]

Electronic Supplementary Material (ESI) for Physical Chemistry Chemical Physics.
This journal is © the Owner Societies 2019

Supplementary Information for: Endohedral metal-nitride cluster ordering in metallofullerene-Ni^{II}(OEP) complexes and crystals: a theoretical study

V. Dubrovin[1], L.-H. Gan[1,2], B. Büchner[1], A.A. Popov*[1], and S.M. Avdoshenko†[1]

[1]Leibniz Institute for Solid State and Materials Research Dresden, 01069 Dresden, Germany
[2]School of Chemistry and Chemical Engineering, Southwest University, Chongqing 400715, China

February 18, 2019

Abstract

The ordering of endohedral clusterfullerenes $Sc_3N@C_{80}$ and $YSc_2N@C_{80}$ co- crystallized with Ni^{II}(OEP) and isolated complexes with Ni(OEP) have been investigated theoretically. Having used multiple orientations of inner clusters inside the cages with Fibonacci sampling, we have described the effect of intermolecular interactions on the orientation of the endohedral cluster.

Keywords: Endohedral metallofullerenes, molecular dynamics, density functional theory, phase space sampling. ■

Contents

1 Geometries 2

2 VASP theoretical settings 2

3 Q-chem theoretical settings 2

4 Initial geometry script 3

5 Stable conformers of $Sc_3N@C_{80}$ and $YSc_2N@C_{80}$ 4

*a.popov@ifw-dresden.de
†s.avdoshenko@gmail.com

1 Geometries

Original structure CRS : originalCRS.xyz (double click here to download XYZ file)

Original structure COS: originalCOS.xyz (double click here to download XYZ file)

All optimized geometries with the energy data:

all COS and CRS minima (right-click here to save TAR archive)

2 VASP theoretical settings

Example 1: VASP INCAR script:

```
PREC = Normal
ENCUT = 400
ISMEAR = 0
SIGMA = 0.1
IBRION = 1
NELM=200
IVDW=11
LREAL=Auto
NSW = 200
EDIFFG = −0.005
ALGO = F
LORBIT=11
LCHARG = F
LWAVE = F
```

3 Q-chem theoretical settings

Example 2: Q-chem4.4 EDA script:

```
$rem
DIRECT_SCF          TRUE
JOBTYPE             EDA
METHOD              PBE
BASIS               6−31G∗
SCF_GUESS           FRAGMO
FRGM_METHOD         STOLL
FRGM_LPCORR         RS_EXACT_SCF
EDA_BSSE            TRUE
DIIS_SEPARATE_ERRVEC 1
$end
```

4 Initial geometry script

Widely used way to generate an evenly spaced mesh on a sphere is to map Fibonacci lattice nodes to the sphere. The Fibonacci sequence a concept well studied in the number theory, its famous 2D representation gives rise to a spiral in the XY-plane with the i-th node given as (i/N, i/ϕ), where N is the node number and ϕ is the golden ratio. Algebraically, from this 2D plane polar angles(θ and φ) can be recovered, so as the spherical coordinates on a unit sphere.

Example 3: Fibonacci set generation script

```
import numpy as np
from ase import Atoms
from ase.io import write, read
import os


def thetaij(v1,v2):
  ab=np.dot(v1,v2)
  a=np.linalg.norm(v1)
  b=np.linalg.norm(v2)
  return np.arccos(ab/(a*b))


def theta_phi(N):
    num_pts = N
    indices = np.arange(0, num_pts, dtype=float) + 0.5
    phi = np.arccos(1 - 2*indices/num_pts)
    theta = np.pi * (1 + 5**0.5) * indices
    return phi, theta


#Setup the system with 120 nodes
ll=0

N=120

#sample points on Fibonacci sphere
phi, theta = theta_phi(N)
```

```
for ip in range(N):
    #read molecule
    mol = read('./original_cos.xyz')
    mol.center()
    pos=mol.get_positions()
    names=mol.get_chemical_symbols()
    cluster=mol[0:4]
    rest=mol[4:len(mol)]
    npos=pos[0]
    #set rotation basis
    v1= pos[1]-pos[0]
    v2= pos[2]-pos[0]
    v3= pos[3]-pos[0]
    v4= np.cross(v1,v2)
    v5= np.cross(v4,v2)
    #rotate molecule to Fibonacci point ip
    cluster.rotate(phi[ni]*180.0/np.pi,v4, center=pos[0])
    cluster.rotate(theta[ni]*180.0/np.pi,v1, center=pos[0])
    cluster1.rotate(90*(-1)**ip,v4, center=pos[0])
    cnew=cluster+rest
    ll=ll+1
    cnew.center()
    #save molecule
    os.system("mkdir_set")
    os.system("mkdir_set/%i" % ll)
    write('set/%i/mol.xyz' % ll, cnew)
    write('set/%i/mol.pdb' % ll, cnew)
```

5 Stable conformers of $Sc_3N@C_{80}$ and $YSc_2N@C_{80}$

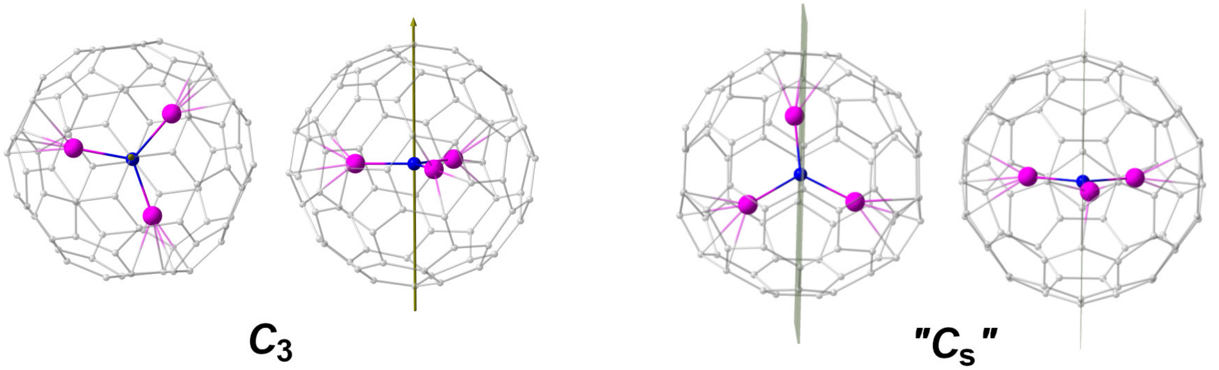

Figure S1: Optimized conformers of $Sc_3N@C_{80}$, each structure is shown in two projections. The lowest-energy conformer has C_3 symmetry (C_3 axis is perpendicular to the cluster plane). The second structure with the relative energy of 2.3 kJ/mole is close to but somewhat deviates from C_s symmetry.

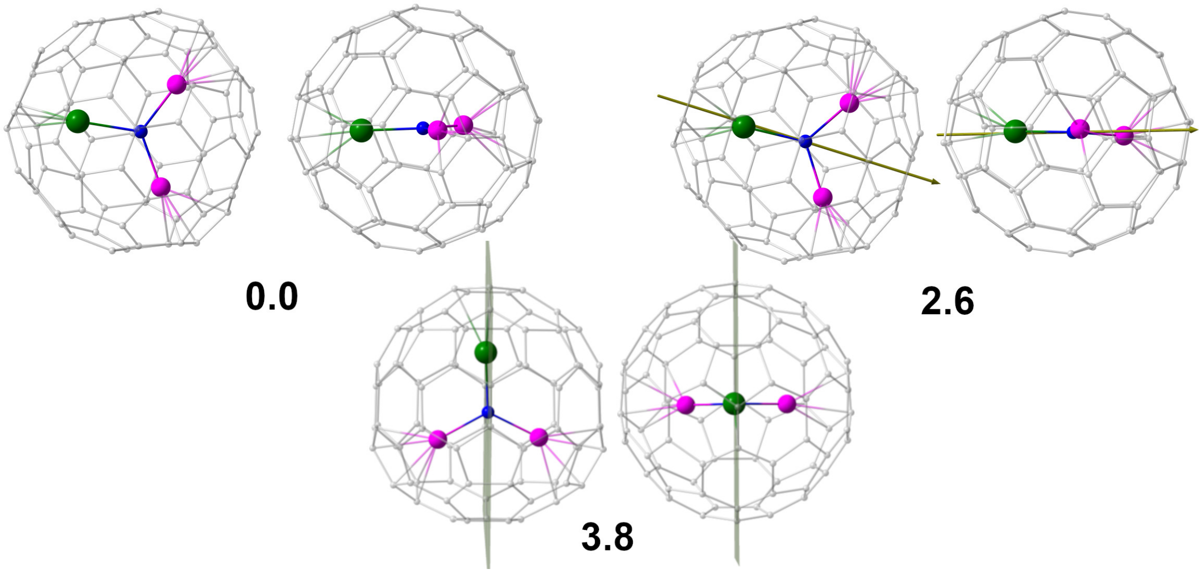

Figure S2: Optimized conformers of $YSc_2N@C_{80}$, each structure is shown in two projections. The lowest energy structure resembles C_3 conformer of $Sc_3N@C_{80}$, in which one Sc atom is replaced with Y. The conformer with the relative energy of 3.8 kJ/mol is similar to "C_s"-conformer of $Sc_3N@C_{80}$, in which the Sc atom close to the symmetry plane is replaced by Y. C_s symmetry in this case is almost rigorous. The conformer with the relative energy of 2.6 kJ/mol is also similar to the C_3 structure but with somewhat rotated cluster, which results in quasi-C_2 symmetry (the axis is also shown in the figure)
